# A Review on Curability of Cancers: More Efforts for Novel Therapeutic Options Are Needed

**DOI:** 10.3390/cancers11111782

**Published:** 2019-11-13

**Authors:** Shuncong Wang, Yewei Liu, Yuanbo Feng, Jian Zhang, Johan Swinnen, Yue Li, Yicheng Ni

**Affiliations:** 1 KU Leuven, Campus Gasthuisberg, Faculty of Medicine, 3000 Leuven, Belgium; shuncong.wang@kuleuven.be (S.W.); yyewei.liu@outlook.com (Y.L.); yuanbo.feng@kuleuven.be (Y.F.); j.swinnen@kuleuven.be (J.S.); 2 Laboratories of Translational Medicine, Jiangsu Province Academy of Traditional Chinese Medicine, Nanjing 210028, China; zjwonderful@hotmail.com; 3 Shanghai Key Laboratory of Molecular Imaging, Shanghai University of Medicine and Health Sciences, Shanghai 201318, China

**Keywords:** cancer treatment, survival, theragnostics, curability and cancer epidemiology

## Abstract

Cancer remains a major cause of death globally. Given its relapsing and fatal features, curing cancer seems to be something hardly possible for the majority of patients. In view of the development in cancer therapies, this article summarizes currently available cancer therapeutics and cure potential by cancer type and stage at diagnosis, based on literature and database reviews. Currently common cancer therapeutics include surgery, chemotherapy, radiotherapy, targeted therapy, and immunotherapy. However, treatment with curative intent by these methods are mainly eligible for patients with localized disease or treatment-sensitive cancers and therefore their contributions to cancer curability are relatively limited. The prognosis for cancer patients varies among different cancer types with a five-year relative survival rate (RSR) of more than 80% in thyroid cancer, melanoma, breast cancer, and Hodgkin’s lymphoma. The most dismal prognosis is observed in patients with small-cell lung cancer, pancreatic cancer, hepatocellular carcinoma, oesophagal cancer, acute myeloid leukemia, non-small cell lung cancer, and gastric cancer with a five-year RSR ranging between 7% and 28%. The current review is intended to provide a general view about how much we have achieved in curing cancer as regards to different therapies and cancer types. Finally, we propose a small molecule dual-targeting broad-spectrum anticancer strategy called OncoCiDia, in combination with emerging highly sensitive liquid biopsy, with theoretical curative potential for the management of solid malignancies, especially at the micro-cancer stage.

## 1. Introduction

Cancer covers a wide spectrum of diseases characterized by uncontrolled and mostly aggressive cell growth, which is driven by down-regulation of tumour-suppressing genes and/or up-regulation of tumour-promoting genes [1]. Although the first cancer case was systematically reported in 1845, it is only in recent decades that in-depth understanding of its biology and pathology has gradually been achieved and tremendous efforts to eliminate cancer have been made [2]. Data from population-based cancer registries estimate a total of 1,762,450 new cases and 606,880 cancer-related deaths in the US in 2019, making it the second leading cause of death [3]. In China, a country with the world’s largest population of over 1.4 billion, 4,292,000 new cancer diagnoses and 2,814,000 cancer-related deaths were reported in 2015, posing a huge burden on both finance and healthcare systems [4]. To counteract the alarming mortality rates, the National Cancer Act of 1971 was launched in the US with the aim to deepen understanding of cancer biology and ultimately prompt the development of more effective cancer therapeutics, which has been, nearly half a century later, upgraded to a newer cancer moonshot funding [5,6]. Benefiting from the advances in clinical therapeutics and management, prolongation in survival for many cancer types has been realized, such as non-small cell lung cancer (NSCLC), hepatocellular carcinoma (HCC), breast cancer, and multiple myeloma, among others [6,7,8,9]. However, it is still premature for us to celebrate the success of curing cancer, as some flaws do exist, e.g., no survival improvement was observed over the past four decades in solid malignancies such as sarcoma and small cell lung cancer (SCLC) [9,10].

The present review, by studying the literature and database, aims to (1) deliver a general landscape of currently available cancer treatments, along with their advantages and disadvantages and future perspective; (2) demonstrate the contribution of these methods to the curability of cancer; (3) quantitatively show the current landscape of cancer diagnosis and prognosis by cancer type, based on data from a population-based database; and (4) put forward a potential liquid biopsy—OncoCiDia strategy, which may revolutionize the future of cancer treatment.

### 1.1. Mortality of Cancer Cells Caused by Therapies

The elimination of cancer cells can be achieved either by complete removal or by induction of cell death. In terms of cell death, which can be either active or passive, active cell death includes apoptosis, autophagy, ferroptosis, activation-induced cell death, mitotic catastrophe, and pyroptosis [11]. The disruption of deoxyribonucleic acid (DNA) structure in the nuclei of cancer cells is a major mechanism for chemotherapy- and radiotherapy-induced apoptosis, and mitotic catastrophe is a molecular event prior to apoptosis [12,13,14]. Additionally, necrosis, as a passive form of cell death following injury and ischemia, can also be induced by chemotherapy, radiotherapy, ablation, and transcatheter arterial chemoembolization (TACE) [15,16,17,18].

### 1.2. Cancer Staging

Heterogeneous progressiveness at diagnosis necessitates a proper classification of cancer stage, which is essential for clinical decision-making and treatment planning. The tumour-node-metastasis (TNM) staging system is the most widely adopted staging system for most cancer types (except for haematological malignancies and brain tumours), and it categorizes patients into four major categories: I, II, III and IV [19]. Stage I patients refer to cases harbouring cancers that are confined within the original organ and are highly curable, whereas stage IV patients are metastatic cases and barely curable. Stage II and stage III patients are with intermediate potentials to be cured, to whom multidisciplinary modalities are applied to maximally prolong their survival [20,21,22]. Due to the futility of single therapeutics in the most advanced cases, cancer cure in these cases could be realized with the “log-kill” model, i.e., surgery or the alike removes a great majority of cancer cells, followed by further chemo- and radiotherapy cleansing; finally, anti-cancer immunity of the patient could be just strong enough to kill the remaining cancer cells [23].

### 1.3. Concepts of Cancer Cure

Literally, to cure refers to eliminate a disease or condition without any relapse. However, the relapsing nature (within months or years) after remission makes the traditional definition of “cure” impractical and rarely mentioned in oncology. The cure of cancer implies not only numerically long survival time, but also equal life expectation between properly treated patients and corresponding cancer-free population. A plausible and empirical criterion for curability is the maintenance of recurrence-free survival for more than 10 years after effective treatments [24]. However, a widely accepted notion for cancer cure in clinic refers to the maintenance of complete remission for five years [25]. Based on this notion, the overall five-year relative survival rate (RSR) for cancer patients was 48.9% in the 1970s and this figure climbed to 69.3% in the 2010s, implying that approximately 30% of cancer patients still fail to be cured [26].

### 1.4. Measurable for Outcomes of Cancer Patients

Assessment of treatment response is pivotal for evaluation of anti-cancer therapies, subsequent treatment planning, and prognosis prediction. Currently available measurable includes survival time/rate and tumour size changes evaluated by imaging. Overall survival (OS), defined as the period from randomization to death, is the gold standard for evaluation of treatment outcome. However, it requires a large sample size as well as long-term follow-up, which is labour intensive and costly. To tackle this and accelerate drug approval, progression-free survival (PFS) or disease-free survival (DFS) were proposed as surrogates and defined as the interval between randomization and objective tumour progression [27,28]. In addition, objective response rate, the proportion of patients with tumour burden reduction classified as either complete remission or partial remission based on response evaluation criteria in solid tumour (RECIST) criteria, correlates well with PFS and serves as an early predictive marker for PFS [29]. The RECIST criteria evaluates therapeutic response by assessment of cancer diameter changes before and after treatment, firstly issued in 2000 [30]. Afterwards, to counteract with the “pseudoprogression” phenomenon during immune checkpoint inhibitors (ICIs) treatment, a modified “iRESCIST” was proposed in 2009 [31].

Besides the above-mentioned measurables at an individual level, epidemiologists and healthcare providers are more concerned about the prevalence of cancer deaths at a population level and thus, cancer-specific survival and RSR are proposed [32]. Cancer-specific survival is the percentage of patients who did not die from the index cancer at a specific time point. Alternatively, the RSR is defined as the ratio between the percentage of cancer patients who survive for a specific period and the percentage of comparable people (usually with the same sex and age) who survive the same period of time [32,33]. Of note, both methodologies have their own limitations, including dependence of accurate classification about cause of death, which is not always possible solely on a basis of clinical evaluation without autopsy, and dependence of comparable referential life table, respectively. And the extent of their difference varies by cancer types and age of diagnosis, with a greater deviation in lung cancer, brain tumour, and the elderly population [34].

## 2. Current Cancer Therapies and Their Performances

**Figure 1:**
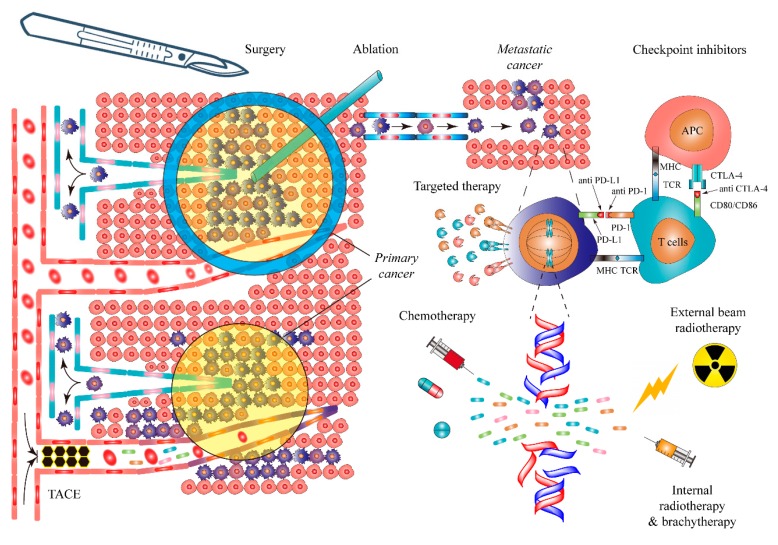
Current major therapeutics for cancer. In the primary site, local treatments, including surgery, imaging-guided interventional procedure, and radiotherapy, can be applied with curative intent. In metastatic disease, surgery, radiotherapy, immunotherapy, targeted therapy, and chemotherapy can be delivered, with palliative or even curative intent. Abbreviations: TACE: transcatheter arterial chemoembolization; APC: antigen-presenting cell; PD-1: programmed death-1; PD-L1: programmed death ligand-1; MHC: major histocompatibility complex; TCR: T cell receptor; CTLA-4: cytotoxic T-lymphocyte-associated protein 4; CD: cluster of differentiation.

Current common cancer treatments include surgery (and its analogous ablation therapies), chemotherapy, radiotherapy, targeted therapy, and immunotherapy (Figure 1). The curative potential for each of these treatments varies and is largely dependent on cancer type, stage, patients’ performance status, and so forth. Surgery alone can be curative in early-stage solid cancers. For instance, the current national comprehensive cancer network (NCCN) guideline recommends radical resection with curative intent and active postoperative surveillance for T1a stage NSCLC [35]. In the hyper-early stage of solid cancers that are sensitive to chemotherapy or radiotherapy, a monotherapy could also be sufficient for cancer cure [36,37,38]. However, a majority of cancer cases are diagnosed at advanced stages in which multimodality strategies are applied, aiming to maximally eliminate cancer cells and prolong patients’ survival. Sadly, long-term survival by these methods are dissatisfactory in practice, and, therefore, the cornerstones of curing cancer still lie in early detection, followed by timely and sufficient treatment. The animal experiments covered by the current review have been approved by the KU Leuven University Ethics Committee (P147/2013).

### 2.1. The Evolving Role of Surgery

Surgery is a major pillar for clinical management of cancer. About 80% of 15.2 million newly-diagnosed cancer patients in 2015 require surgery across the globe and by 2030 this figure will increase to 45 million [39]. Besides preventive, diagnostic, and cosmetic purposes, surgery contributes substantially to the cure of solid cancer at their infancy by removal of cancer tissue and lymph node. It has served as the only curative therapy for most solid tumours at an early stage, such as oesophagal cancer, gastric cancer, and colorectal cancer (CRC), to mention only a few (Table 1). However, a majority of cancer cases were diagnosed at locally advanced or even metastatic stages, limiting the applicability of curative surgery. The development of oncological surgery mainly focuses on decreasing its invasiveness that, on a basis of equivalent anticancer efficacy, may help minimize perioperative complications and postoperative side-effects, and ultimately improve patients’ quality of life. Laparoscopic surgery has shown equivalent anticancer efficacy and lower incidence or intensity of complications, compared with open surgery in gastric cancer and colorectal cancer [40,41]. However, critics indicate that high-quality evidence from well-designed randomized clinical trials with sound methods is needed to justify the routine implementation of laparoscopic gastrectomy [42]. Furthermore, laparoscopic surgery in pancreatic or periampullary cancer and cervical cancer was associated with an inferior oncologic outcome compared with open surgery [43,44,45,46]. Endoscopic surgery has been applied in early oesophageal cancer, gastric cancer, and colorectal cancer in carefully selected patients in expert centres, such as T1a gastric cancer without lymph node metastasis [47,48,49,50]. Despite versatile flexibility of robotic surgery, its associated survival benefit and cost-effectiveness have been disputed, especially in mastectomy and other cancer-related surgeries [51,52]. Concerns about minimally invasive surgery include long learning curve, dependence of centralization of cases in hospital with high volume of surgeons, limited eligibility (only for highly-selected patients), and higher cost [53,54,55,56]. As the alternatives to the above-mentioned open and minimally invasive surgeries, which make the entire tumour (and a layer of surrounding tissue as a safety margin) physically excised from the patient, a series of minimally invasive tumour ablation techniques have been developed. These virtual surgical techniques (Table 1) instantly kill the tumour (also with an intended 5–10 mm peritumoural safety margin) in situ without actual tumour removal by local delivery of either lethal temperatures such as hyperthermal radiofrequency ablation and hypothermal cryotherapy, or caustic chemicals such as absolute ethanol and acetic acid with resulted tissue necrosis [57,58,59]. Likewise, imaging-guided interventions such as TACE have been often applied for the treatment of mainly hepatic malignancies with palliative expectation [60,61].

### 2.2. The Pros and Cons of Radiotherapy

Radiotherapy, including external beam radiotherapy (EBRT), internal radiotherapy, and brachytherapy, has been used in about 50% of cancer patients and exerts its anticancer activities by ionizing radiation, which structurally damage DNA or other macromolecules, resulting in mainly apoptosis in all exposed cells (Table 1) [62]. Radiotherapy is limited by complexity of radiobiology, difficult achievement of perfectly conformal dose distribution, and risk of secondary malignancy [63]. EBRT can be classified as different subtypes, based on the emitted particles: photons, electrons, and particles (proton, neutron, and heavy ion) [64]. Heavy ion refers to particles having one or more units of electric charge and a mass exceeding that of the Helium-4 nucleus (alpha particle) [65]. Photon radiotherapy, currently the most frequently used subtype, can generate free radicals and cause single strand DNA damage [66]. Electron radiotherapy has a short penetration, after which the energy drops sharply and therefore it is mostly used in superficial cancer [67]. Particle radiotherapy, delivering high-energy radiation, may form a dose peak near the end of penetration, namely Bragg peak, which enables higher dose in cancer tissue, induction of double strand DNA damage in a less oxygen-dependent manner, and better sparing of surrounding normal tissue [68]. Particle radiotherapy has become increasingly popular, especially over the past two decades [69]. Until the end of 2016, 174,512 patients received particle therapy globally, with 149,345 receiving proton therapy and 21,580 receiving carbon ion therapy [70]. However, widespread use of particle radiotherapy was halted by its tremendous cost and a paucity of solid clinical evidence [71]. EBRT is usually administrated in combinatory settings in most cancer cases, except for localized cancer cases with high sensitivity like lymphoma and seminoma. To achieve a possible curative effect with radiotherapy, a typical requirement is to reach a cumulative radiation dose ranging from 50–80 Gy for most cancer types, with 30 Gy in lymphoma [72,73]. The development of EBRT mainly focuses on two directions: better sparing of normal tissue by precise delivery and enhanced cancer control. The preciseness of delivery depends on the accurate delineation of the extent of tumour, developing from computed tomography (CT) to positron emission tomography (PET)/CT, a method incorporating metabolic information [74]. Currently, many linear accelerators are equipped with CT imaging, which can confirm the tumour location before delivery, namely image-guided radiation therapy [75]. Intensity-modulated radiation therapy (IMRT), a form of precision radiotherapy, was developed to enable the formation of a high-dose region with conformal complexity and proximity to vital tissue; an analog to IMRT is volumetric-modulated arc therapy, which requires short delivery time. Additional technical developments to achieve both higher conformity and possibly greater anti-cancer efficacy include protons and heavy ions therapy [76,77]. Enhanced cancer control can be achieved using various methods. Hypofractionation, namely stereotactic body radiation therapy, was proposed for its additional indirect effects like vascular collapse and immune effects in intracranial tumours; its application in extracranial tumours, namely stereotactic ablative radiotherapy, is currently being explored [78,79,80,81,82]. Additionally, combination therapies have strived to improve efficacy—the concurrent chemoradiotherapy, which is the cornerstone for a wide spectrum of cancers, is the most successful one. Radiotherapy may synergistically act with immunotherapy by releasing tumour antigens and a modulating microenvironment that facilitate recruitment of immune cells [83]. Another potential combination is with nanoparticles (NP). Gold NP can enhance radiosensitivity physically (producing photoelectron, Auger electron and low energy secondary electron), chemically (radical formation and chemical sensitization), and biologically (cell cycle disruption, oxidative stress, and DNA repair inhibition) [84,85,86]. For the DNA repair inhibition effect, nanoparticles exert an inhibitory effect by electric field generated from ionization of nanoparticles in the irradiated tissue [87]. NP, which can decompose H_2_O_2_ to generate O_2,_ may help relieve the hypoxia, and enhance the immunogenicity of radiotherapy [88,89]. Currently, two NPs are under clinical trials: NBTXR3, a hafnium-based intratumourally administered NP and AGuIX, a gadolinium-based intravenously administered NP [90,91,92]. The addition of NBTXR3 to radiotherapy may help improve the pathological complete response rate of locally advanced soft-tissue sarcoma (16% vs. 8%); the first clinical trial of AGuIX (NCT02820454) is finished, awaiting results [93,94]. Internal radiotherapy, which consists of radioactive isotope and radiopharmacy, was delivered based on intrinsic enrichment, intake of ^131^I by thyroid cancer, and ^223^Ra by bone cancer lesion; or artificial enrichment by intercalating radioactive isotopes with a receptor for tumour biomarker like CD20 antibody in Zevalin [95,96,97]. Brachytherapy was given directly or via a catheter implanting radioactive isotopes in or next to the cancer site, which produces high-energy radiation directly to the tumour [98]. Moreover, it shows better sparing of surrounding tissue for a sharp dose fall-off outside its limited penetration zone and a higher radiation dose in the cancer site, compared with EBRT [98]. Brachytherapy for breast cancer, prostate cancer, and cervical cancer is mainly preformed with ^125^I, a low dose rate isotope, which emits photons with energy up to 35.5 keV by gamma decay and X-rays of energy between 27.2–31.7 keV by transition [99]. However, radiotherapy may also affect normal cells and induce side-effects due to imperfect conformal delivery, especially for fast-proliferative cells, like intestinal epithelia, bone marrow blood stem cells, etc. [100,101,102]. Additionally, patients are associated with a slightly higher incidence of secondary malignancy five years after receiving radiotherapy, especially those treated for breast cancer, mediastinal B-cell lymphoma, seminoma, prostate cancer, cervical cancer, and endometrium cancer [103,104,105,106,107]. Similarly, radiotherapy by radioactive iodine (RAI) for thyroid cancer could be associated with a higher incidence of secondary malignancies, especially in cases with a cumulative RAI dose over 150 mCi [108]. RAI treatment of well-differentiated thyroid cancer showed 0.5% risk of developing acute and chronic myeloid leukemia [109].

### 2.3. The Contribution of and Concerns about Chemotherapy

Chemotherapy aims to eliminate cancer cells or inhibit their growth by altering abnormal cellular proliferation and metabolism, which are spectacular hallmarks of malignancies (Table 1). Chemotherapy is limited by a low response rate (except for a few sensitive cancer types), systematic side-effects, and risk of a secondary malignancy. The therapeutic efficacy of chemotherapy varies among different cancer types and satisfactory therapeutic effect is only shown for a limited spectrum of malignancies, including acute leukemia, Wilm’s tumour and Ewing’s sarcoma in children, and choriocarcinoma, lymphoma, endometrial cancer, and seminoma in adults. The general contribution of curative and adjuvant chemotherapy to five-year survival in adults is less than 2.5%, providing an extra survival benefit of merely three months [110]. The addition of adjuvant chemotherapy was proposed to eliminate the remaining cancer cells after radical surgery and reduce the risk of postsurgical relapse; its associated survival improvement was practically observed in early stage epithelial ovarian cancer, esophagus cancer, etc. [111,112]. However, the survival benefit is absent in ypTis-2N0 rectal cancer and stage II colon cancer [113]. For advanced stage cancer, the complete remission rate for chemotherapy is generally low (7.4%), regardless of cancer type and drug regimen [114]. Moreover, most of these cases relapse shortly after treatment. For instance, approximately 90% of metastatic NSCLC and pancreatic cancer patients progressed within 15 months after chemotherapy, with 90% of metastatic gastric and advanced esophagogastric cancer progressing within 24 months [115,116,117,118]. The contribution of chemotherapy to the survival of advanced hepatocellular carcinoma and pancreatic cancer is marginal [115,119]. Escalated chemotherapy is not necessarily associated with improved survival. The addition of cetuximab to postoperative chemotherapy for Ki-ras2 Kirsten rat sarcoma viral oncogene homolog (KRAS) exon 2 wild-type colorectal cancer was associated with a shorter PFS [120]. In contrast, the de-intensified regimen can help achieve equivalent anti-cancer efficacy in selected low-risk patients with breast cancer, colon cancer, or human papillomavirus–associated oropharyngeal squamous cell carcinoma [121,122,123,124]. The main hindrance to limited anti-cancer activity in chemotherapy is heterogeneous sensitivity in the diverse cancer cell population: indolent or insensitive cancer cells is the major resource of relapse [125]. Heterogeneous sensitivity can also be observed on an individual level: (1) response rate for solid tumours is generally lower than 50%, with 20–30% in NSCLC [126]; (2) highly responsive patients can be identified by biomarkers, including DNA damage immune response assay, promoter methylation for oesophageal adenocarcinoma patients, and 21-Gene Recurrence Score Prognostic Assay in early breast cancer patients [38,127,128,129]; and (3) higher response rate of pemetrexed in lung adenocarcinoma [130]. Accordingly, the role of chemotherapy in less-responsive cancer types has been challenged by targeted therapy or immunotherapy. In completely resected stage II-IIIA epidermal growth factor receptor (EGFR)-mutant NSCLC, adjuvant gefitinib is associated with better DFS than platinum-based chemotherapy [131]. In metastatic NSCLC, the first-line chemotherapy regimen remained a platinum-based regimen for decades, with a plateau response rate between 20% and 30% [126]. Currently, in patients with druggable targets (EGFR, anaplastic lymphoma kinase (ALK), and so on), targeted therapy has been recommended as the first line therapy [132,133]. More recently, the role of chemotherapy as first line treatment in mutation-negative advanced NSCLC was challenged by dual blockade of cytotoxic T-lymphocyte-associated protein 4 (CTLA-4) and programmed death 1 (PD1) (median OS: 14.9 vs. 17.1 months) [134].

In addition, chemotherapy is associated with a higher risk of a secondary malignancy, with increased risk of bone tumours and leiomyosarcoma after addition of an alkylating agent to radiotherapy for treatment of hereditary retinoblastoma [135]. The side-effects of chemotherapy are often systemic, especially on organs with rapidly growing cells, such as intestinal epithelia, bone marrow blood stem cells, and hair follicle cells [136].

### 2.4. The Contribution of and Concerns about Targeted Therapy

Targeted therapy was developed on the basis of in-depth understanding of cancer biology, and is frequently used in NSCLC, lymphoma, breast cancer, gastric cancer, and colorectal cancer, among others (Table 1). The development of targeted therapy in NSCLC has changed the first-line treatment regimen for druggable mutation-positive patients: from platinum-based chemotherapy to targeted therapy in advanced and post-operative NSCLC [131,132,133]. The addition of rituximab to chemotherapy in CD20+ diffuse large B cell lymphoma significantly prolongs patients’ DFS and OS [137]. However, the ‘preciseness’ in themselves are Achilles’ heel to some extent: limited eligibility and resistance due to evolution of cancer cell population from sensitive to insensitive ones. For instance, 50% of Asian patients and 10–15% of Caucasian patients with lung adenocarcinoma are EGFR mutation-positive, with 5% of NSCLC being ALK positive and 1% of NSCLC patients being ROS1 positive [138,139,140]. Moreover, treatment escalation by a newer generation of drugs is an inevitable but a rarely possible way, except for EGFR-mutated NSCLC, wherein acquired T790M mutation after administration of the first generation tyrosine kinase inhibitor (TKI) can be successfully targeted by osimertinib, the third generation TKI [141]. Additionally, therapies that target the growth of tumour blood vessels, rather than cancer cells are also available [142]. The signaling axis for angiogenesis consists of pro-angiogenetic molecules, corresponding receptors, and post-receptor signaling pathways, which jointly promote endothelial cell proliferation, migration, survival, and ultimately angiogenesis [143]. This cascade reaction can be inhibited at different levels, with Bevacizumab targeting vascular endothelial growth factor, Ramucirumab targeting vascular endothelial growth factor receptor-2, and TKI (cabozantinib, lapatinib, and sorafenib, to mention a few) targeting post-receptor signaling pathways. However, the major limitations for these strategies are the lower response rates, ranging between 2–30%, rare but fatal complications like perforation and haemoptysis, and acquired resistance [143]. In addition, another group of drugs, namely vascular-disrupting agents (VDAs), can hinder the growth of cancer by either disrupting pre-existing blood vessels in cancer stroma or by having a direct cytotoxic effect on cancer cells [144]. However, current clinical trials have demonstrated its unsatisfactory competence in both single and combinatory settings for advanced cancers [145,146,147]. The bottleneck problem with VDAs appears to be incomplete tumour necrosis with remnant viable cancer cells that cause tumour regrowth. However, the new OncoCiDia strategy seems to be able to tackle such a bottleneck problem (see details in Section 5).

### 2.5. The Contribution of and Concerns about Immunotherapy

Lastly, immunotherapy, including cellular therapy, cytokines, or ICIs aims to treat cancers by increasing or restoring anticancer immunity (Table 1). Successful sporadic cure has been reported in chimeric antigen receptor T cells therapy and ICIs; however, the response rate is low, unpredictable, and vulnerable to other biological factors. The ICIs are gaining increasing popularity for their uniqueness in durable response and high response rates in some relapsed cancers. Ipilimumab, the first commercial monoclonal antibody targeting CTLA-4, was shown to offer a superior survival benefit for metastatic melanoma in 2011, compared with peptide or combinatory dacarbazine chemotherapy [148,149]. More strikingly, approximately 15% of patients showed a durable response more than 10 years after therapy discontinuation, which distinguishes immunotherapy from conventional therapies [150,151,152]. In addition, immunotherapy targeting the PD1/PDL1 axis has been receiving approval since 2014 for second-line or first-line therapies for an increasing number of malignancies, including melanoma, lymphoma, NSCLC, renal cell cancer, head and neck squamous cell cancer, bladder cancer, liver cancer, esophagogastric junction cancer, and micro-satellite unstable cancer of any origin [153,154]. The dual blockade by both CTLA4 and PD1/PDL1 yield improved survival in melanoma and NSCLC [134,155]. However, the response of ICIs varies among cancer types, with high response rates in melanoma, NSCLC, Hodgkin’s lymphoma, Merkel cell carcinoma and microsatellite instability-high CRC, and with low response rate and marginal survival benefit in SCLC, renal cell carcinoma, and head and neck cancers [156]. More importantly, it is impossible to predict and identify patients that could potentially benefit from this therapy. Although biomarkers, including tumour mutation burden, programmed death ligand 1 (PD-L1) expression, lymphocyte infiltration rate, and tumour-immune phenotypes, were identified, their clinical efficacy for predicting treatment response remains unconfirmed and controversial [157,158]. Furthermore, the anticancer efficacy of ICIs is vulnerable to prior application of antibiotics, with significantly worse OS in patients receiving antibiotics (2 vs. 26 months) [159]. Additionally, hyperprogression is a deleterious effect of checkpoint inhibitors, characterized by accelerating cancer growth with an incidence rate of 9% (12/131) [160]. More importantly, ICIs are associated with fatal toxicity effect in 0.3–1.3% patients, as reported more recently [161].

## 3. Curability by Cancer Type and Stage

**Figure 2:**
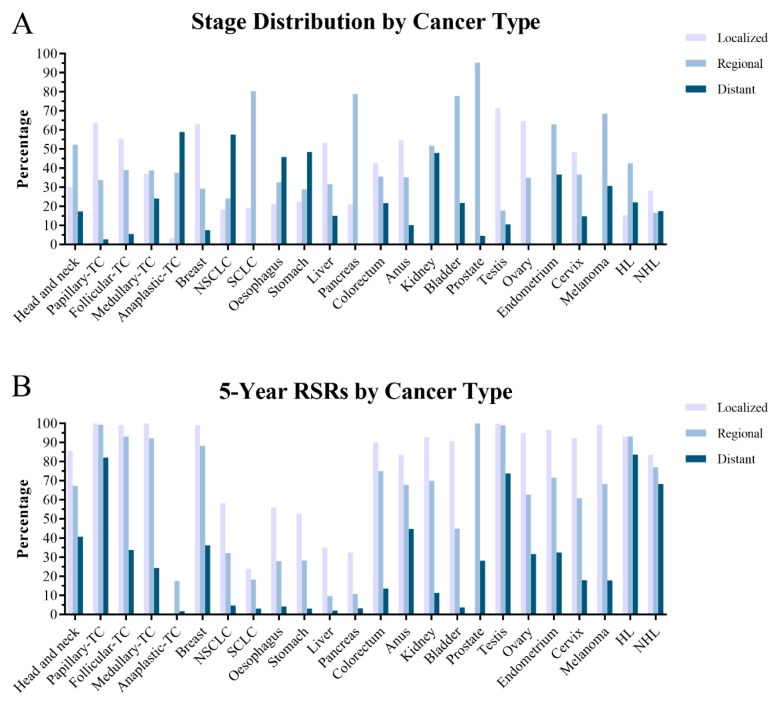
The distribution of stage (**A**) and corresponding five-year relative survival rates (**B**) by cancer types, based on cases diagnosed in 2010 in nine SEER registries. All data here are accessed from the Surveillance, Epidemiology, and End Results (SEER) Program (www.seer.cancer.gov) SEER*Stat Database: Incidence - SEER 9 Regs Research Data, November 2018 Sub (1975–2016). Note: Localized and regional prostate cancer cases are merged as localized/regional cases.

To quantitatively show what we have achieved with the currently available diagnostic methods and treatments for different cancers, we present the stage distribution and corresponding five-year RSR in patients diagnosed in 2010 from the nine registries in the large population-based surveillance, epidemiology and end results (SEER) database [213]. Table 2 summarizes the curative potential by cancer type based on currently available therapeutics. To ensure the comparability and consistence of stage in different cancer types, we adopted the staging system provided in the SEER database, which contained three categories, including localized, regional, and distant. Here, localized cases refer to cancer lesion confined within its originated organ, with regional cases referring to cancer lesion spread to adjacent tissue (without metastasis) and distant cases referring to metastatic cases. Here, most patients were diagnosed at an advanced stage (regional or distant) in a majority of cancer types. Thus, early diagnosis by increased public awareness, widespread screening protocol, and development of more sensitive and discriminative detection methods may help change the scenario [214]. The prognosis varies among different cancer types with a five-year RSR of more than 80% in thyroid cancer, melanoma, breast cancer, and Hodgkin’s lymphoma. However, the prognosis for SCLC, pancreatic cancer, HCC, oesophageal cancer, acute myeloid leukemia, NSCLC, and gastric cancer is still dismal, with a five-year RSR ranging between 7% and 28%, emphasizing further endeavours in combating cancers (Figure 2). Some new drugs merely provide marginal survival benefit: 14 novel regimens approved for solid tumours by European Medicines Agency (EMA) are associated with a median OS benefit of 1.2 months, with a median OS benefit of 2.1 months for 48 new regimens approved by FDA between 2002 and 2014 [215,216]. It is estimated that development in treatment options explains only the 20% increase in five-year survival—from 49–68% over 40 years—whereas development in early diagnosis may have contributed much more [217,218,219]. Moreover, among 32 new drugs approved by EMA between 2014 and 2016 on the basis of 54 trials, only 10 randomized trials measured OS, with 19 randomized trials harbouring high risk of bias [220].

## 4. Cancer Screenings and Their Pros and Cons

In parallel to the development of therapeutics, the development of screening also contributes to the improvement of cancer survival by promoting early diagnosis. Currently, the U.S. Preventive Services Task Force recommends screening in breast cancer by biennial mammogram for persons aged 50 and 74 years; cervical cancer by Pap test and/or HPV test for persons aged over 21 years; CRC by stool test, endoscopy or CT colonography for patients with aged between 50 and 75 years or earlier for high risk patients; and lung cancer by annual low dose CT for heavy smokers or persons aged between 55 and 80 years [245,246]. The goal of cancer screening is to achieve early diagnosis of cancer, which is destined to progress, and ultimately to prolong patients’ survival by constantly emerging treatments. Screening in colon cancer and cervical cancer yields successful results, with a 70% reduction in mortality in cervical cancer and either 26% (data based on 155,000 patients from a USA trial) or 31% (data based on 170,000 patients from a UK trial) reduction in mortality in colon cancer [247,248,249].

However, screening may associate with overdiagnosis—detection of asymptomatic or indolent cancers that are not deemed to cause harm and therefore no active treatment is needed. Overdiagnosis is prevalent in breast cancer, prostate cancer, CRC, thyroid cancer, and melanoma [246,250,251,252,253]. The estimated overdiagnosis rate for breast cancer by mammography is 25%, with 50–60% in prostate cancer by prostate-specific antigen (PSA), and 13–25% in lung cancer by low-dose CT [254,255,256]. Mammography can detect more early-stage breast cancer but fails to induce commensurate incidence reduction in advanced disease as well as mortality in the population [251,252]. In addition, indolent breast tumours (slowly growing and estrogen-negative) are more easily detected by screening, a phenomenon termed length bias [257,258,259]. In terms of prostate cancer, screening by PSA is abandoned due to the high false-positive rate about 70% and a false negative rate of 15% [260,261,262]. Moreover, approximately half of the prostate cancer is indolent and silent, as it can be detected by autopsy in 36% of white men and 51% of black men who died from other causes [263,264]. In CRC, a surveillance study by colonography shows a diverse natural history of small polyps, with only 22% of them growing, 50% being stable, 28% shrinking, and 10% completely regressing [265]. Moreover, polyps were discovered in 32% of participants aged 60 years and more than 50% in older individuals from an international, population-based screening study, compared with the much lower risk of developing CRC (approximately 5%) [266,267]. The futility of screening lies in cancer heterogeneity, i.e., not every tumour will ultimately progress or proceed at a pace rapid enough to ensure that early treatment can yield survival benefit during the limited life of a human. Thus, a more discriminative method based on a deeper understanding of cancer biology may better identify patients harbouring a rapidly-progressing tumour that necessitates timely treatment. Recently advocated high sensitivity liquid biopsy techniques are examples of such efforts [268,269,270].

## 5. A Newly Proposed Broad-Spectrum Anti-Cancer Strategy Based on a Dual Stroma-Targeting Approach: Orchestrating with Liquid Biopsy

Unlike therapies that are aimed at heterogeneous cancer cells, we developed a strategy called OncoCiDia, which targets cancer stroma components, which are more homogeneous and less mutational than cellular components [271]. The OncoCiDia strategy first applies a VDA that targets the misstructured endothelia of tumour blood vessels and induces massive (but never complete) ischemic tumour necrosis in virtually all solid cancers. However, following VDA injection, active angiogenesis and thereby cancer relapse are triggered due to cellular response to hypoxia, a pathophysiological phenomenon on which three winners shared the 2019 Nobel Prize for physiology and medicine [185]. To tackle this, in practice overnight after VDA administration, the patient is given radioactive ^131^I labeled necrosis-avid hypericin, which selectively sticks to the necrotic tumour site and constantly irradiates the remaining cancer cells using high-energy beta particles; meanwhile, the emitted gamma rays facilitate scintigraphy imaging. OncoCiDia represents a one-stop-shop theragnostic strategy: visualization, therapeutics, and monitoring radiation distribution, as compared to the ^177^Lu-Dotatate strategy, where a pretreatment PET scan is needed to identify optimal patients and predict treatment response [272,273]. A high percentage of the injected dose per gram of tissue (ID%/g, median: 3.13%; IQR: 2.92–3.97%) of ^131^I-hypericin was observed in the tumour site eight days after injection, constituting a cumulative radiation dose of about 5000 Gy, higher than that of antibody-based immunoradiotherapy with a concentration of 0.001–0.01% and a cumulative radiation dose of 15 Gy [271,274]. Compared with ^177^Lu-Dotatate excreted via the kidney, excretion of ^131^I-hypericin by liver-bile duct-intestine, organs with a higher tolerance dose than that of the kidney, appears to be safer [275,276,277,278,279]. Moreover, targetability of OncoCiDia seems superior to that of ^177^Lu-Dotatate, which may go ‘off target’ in the kidney and spleen due to the expression of somatostatin receptors [271,280,281]. Currently, early phase clinical trials of OncoCiDia are ongoing in both veterinary and human patients [282,283].

OncoCiDia has a few unique features: First, it provides precise but wide-spectrum therapeutics, compared with conventional molecule-based targeted therapies that merely focus on patients with a druggable target. For instance, 17% of lung adenocarcinoma shows sensitive EGFR mutation, with 7% showing ALK mutation and 3% showing MET mutation [223]. Moreover, mutation of ROS1, BRAF, RET, NTRK1, PIK3CA, and MEK1 occur in only 1% of lung adenocarcinoma [223]. More importantly, in eligible patients, due to cancer heterogeneity, cancer evolution (and subsequent resistance) ultimately occurs on shifting from a sensitive cell population to an insensitive one [284]. OncoCiDia, which targets the abnormal endothelia of tumour blood vessels, a target common to nearly all solid cancers, can substantially benefit more patients, and its efficacy is less likely affected by cellular heterogeneity. Second, OncoCiDia enables real-time monitoring of the accumulation of radiation in cancer lesions. Third, the cost of conventional targeted therapy is enormous, as a tiny proportion of eligible patients covers the cost for drug development. Forth, the response rate of conventional targeted therapy is modest in target-positive patients, with 47% in HER-2 mutated gastric cancer [285], 71.2% for gefitinib in EGFR-mutated NSCLC [133], 71% for osimertinib for EGFR T790M mutated NSCLC [141], etc.

**Figure 3:**
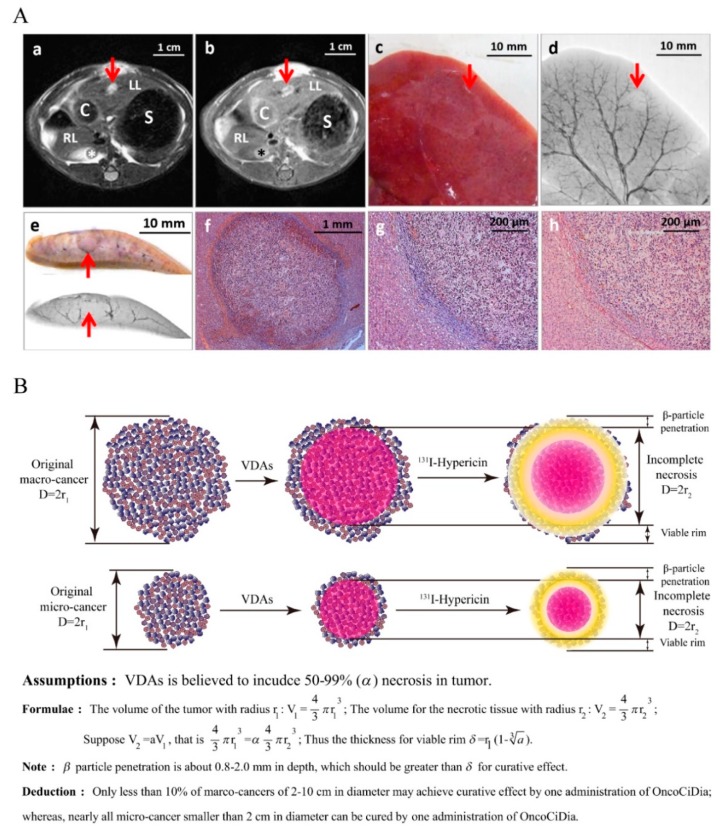
(**A**) A representative example of rats with liver implantation of rhabdomyosarcoma (R1) 12 h after CA4P treatment. This micro R1 tumour measures 3.3 mm and 2.5 mm in long and short axis diameters, respectively. a: on T2 weighted transverse MRI, an oval hyperintense liver lesion (arrow) appears in the left liver lobe (LL); RL, right liver lobe; S, stomach; and C, colon. b: 15 min after contrast agent Gd-DOTA administration, left liver (LL) lesion is enhanced with a central dark region (arrow) suggestive of necrosis; RL, right liver lobe; S, stomach; and C, colon. c: liver specimen containing the micro R1 tumour (arrow) that is too small to be seen from the surface. d: corresponding microangiography shows the lesion as a filling defect suggestive of necrosis (arrow). e: the lesion (arrow) can be traced on the liver section (upper) and corresponding microangiography (bottom). f: low power HE stained microscopy reveals massive and partial hemorrhagic tumour necrosis with tissue reaction and possible tumour residues at the periphery of this virtually hypo- to avascular R1 tumour. g: higher power HE stained microscopy clearly depicts the central necrosis and peripheral few layers of viable R1 tumour cells without noticeable intratumoural vasculature. h: corresponding immunohistochemical CD34-PAS dual staining microscopy confirms the findings with HE staining. (**B**) A proposed curative OncoCiDia strategy with mathematical algorithms. In early-stage cancer, after the induction of nearly complete necrosis by systemic administration of a VDA, subsequently administered ^131^I labelled hypericin can precipitate in tumour necrosis and the emitted beta particles can fully cover the remaining cancer cells particularly in small solid malignancies or micro-cancers. The upper row simulates macro-cancers, with the lower row simulating micro-cancers.

Besides, the curative potential of OncoCiDia in early cancers has been preliminarily implied by the successful induction of nearly complete necrosis by CA4P in primary [286] and secondary micro-cancers (Figure 3A). Here, we propose an updated hypothetical utility of OncoCiDia. If cancer can be detected at an early stage, i.e., micro-cancers of 2–5 mm in diameter undetectable by current imaging modalities but likely detectable by newly emerging supersensitive liquid biopsy techniques [269,270], the remaining cancer cells after VDAs in such micro-cancers can be eradicated under the full coverage of beta radiation emitted by ^131^I with a 2 mm penetration distance (Figure 3B).

Liquid biopsy provides comprehensive information on the diagnosis, treatment response monitoring, and prognosis prediction by analyzing circulating tumour cells or cancer cell-derived fragments, especially DNA (ctDNA) [287]. With regard to early diagnosis, the CancerSEEK panel, incorporating detections of tumour biomarkers and ctDNA, achieves an overall median sensitivity of 70% and specificity of ≥ 99% in diagnosing cancers of the ovary, liver, stomach, pancreas, esophagus, colorectum, lung, and breast [268]. The sensitivity for stage I liver cancer is 100%. Liquid biopsy on urine can empower early diagnosis of bladder cancer and recurrence surveillance [288,289]. In addition, liquid biopsy may also help identify minimal residual disease (MRD) in solid tumours before conventional imaging test does. MRD is a major resource for latent late recurrence, with 13% of T1N0 hormone receptor-positive breast cancer developing recurrence 20 years after therapy with curative intent [290]. In addition, recent reports have confirmed the metastasis of breast cancer and colorectal cancer even at earlier stages [270,291,292,293]; thus, OncoCiDia and liquid biopsy could synergistically play a role in such scenarios. This may open a new horizon for cancer management.

## 6. Conclusions

Over the past decades, great progress has been made in cancer diagnosis and therapeutics, which helps prolong survival in most cancer types. However, prognosis in some cancer types is still dismal and has only improved marginally over time. Therefore, early diagnosis is pivotal in improving the cure rate, and screening of the high-risk population seems to be a practical request. Given the ultra-sensitive characteristics and successfulness in detection of early cancer by liquid biopsy, the combinatory use of this method and the proposed OncoCiDia approach may be a non-invasive and curative or preventive strategy, particularly for patients with micro-cancers.

## Figures and Tables

**Table 1:** Summary of the curative potential of currently available cancer therapeutics.

Cancer Therapy *	Mechanism	Curative Potential	Example	Limitations
Surgery				
Open surgery	Physical removal of cancer, adjacent tissue, and involved lymph nodes	For early solid cancer, +++	Early NSCLC [162], HCC [163], renal cancer [164]	Surgical injury [165], cancer dissemination [166]
Laparoscopic surgery	Same as above	Same as above	Same as above	Surgical injury [165], cancer dissemination [166], dependence of centralized expert surgeons [53]
Robotic surgery	Same as above	Same as above	HCC [167], prostate cancer [168]	Same as above, imperfectly confirmed efficacy
Endoscopic surgery	Same as above	Same as above	Early GI cancer [169,170]	Possible second surgery [171], perforation [172]
Interventions				
Ablations	In situ necrotizing cancer and adjacent tissue by local hyperthermal ablation, cryotherapy, or absolute ethanol injection.	For early eligible cancer, +++	HCC [173], renal cancer [174], lung cancer [175]	Often incomplete ablation [176], injury to adjacent tissue [177,178]
TACE	Embolization of cancer supplying artery combined with local chemotherapy	−/+	HCC [179]	Often incomplete cell death [179]
Chemotherapy				
Direct	Alteration of DNA synthesis and structure or cytoskeleton	For chemotherapy sensitive cancer, +++	Early lymphoma [180], ALL [181], seminoma [182]	Pancytopenia, nausea, infertility, neuropathy [136], secondary cancer [183]
Indirect	Immunomodulation, vascular disrupting effect	−/+	NA	Venous thrombosis [184], recurrence [185]
Radiotherapy				
External beam	Alteration of DNA structure via radicals	For radiotherapy sensitive cancer, +++	Early lymphoma [186], NPC [187], +++	Unintentional destruction along entrance channel [188], secondary cancer [189]
Radioiodine	Same as above, for thyroid cancer with iodine intake	For thyroid cancer with iodine intake, ++	Thyroid cancer [108]	Side-effects [190], secondary cancer [109]
Radiopharmacy (Lutetium 177)	Same as above	−/+	NA	Side-effects [191], secondary cancer [192]
Brachytherapy	Same as above	−/+	NA	Side-effects [193], secondary cancer [194]
Targeted therapy				
Direct	Inhibition of signaling pathway, ADCC for monoclonal antibody	−/+	NA	Acquired resistance [195]; narrow spectrum of optimal patients [196]
Indirect	Anti-angiogenesis	−/+	NA	Acquired resistance [197], low response rate [198], rare but fatal side-effects [198,199,200]
Immunotherapy				
Immune checkpoint inhibitors	Restore anticancer immunity	Only possible in responded cases (about 10%) [201], +	Melanoma [148]	Unpredictability of response [158], rare but fatal side-effects [161]
Cellular immunotherapy	Elimination of cancer cells by immune cells with or without engineering	−/+	Leukemia [202]	High cost [203], severe side-effects [204]
Bone marrow transplantation	Elimination of cancer cells by intensive chemotherapy and graft-versus-leukemia effect	Yes, for high-risk haematological cancer [205], +++	Leukemia [206]; lymphoma [207];	High mortality rate (5%) [208] and extensive post-transplantation care [209]
Endocrine therapy	Inhabitation of growth by altering hormone signaling	Unknown ^#^, for hormone receptor positive patients, ++	Prostate cancer [210], breast cancer [211]	Secondary cancer [212]

Abbreviations: ADCC: antibody-dependent cellular cytotoxicity; ALL: acute lymphoid leukemia; DNA: deoxyribonucleic acid; HCC: hepatocellular carcinoma; NSCLC: non-small cell lung cancer; NA: non-applicable; GI: gastrointestinal; ALL: acute lymphoid leukemia; NA: non-applicable; NPC: nasopharyngeal carcinoma; TACE: transarterial chemoembolization. * We refer to the therapies in each category with curative intention; ^#^ hormone is often used in combinatory settings and therefore its role in single-use remains unclear. -/+: unlikely but possible; +/-: possible but unlikely; + limited curative potential, only possible in some cases; ++ contribute to the cancer cure in combination; +++ with curative potential.

**Table 2:** Summary of curative potential by cancer type and stage based on currently available cancer therapeutics.

Cancer Type	Current Treatment	Curative Possibility, 5-Year RSR	Curative Methods	Stages Distribution (Localized, Regional, Distant) ^‡^	5-Year RSR by Stage ^‡^
Head and Neck	Surgery, radiotherapy, chemotherapy, targeted therapy, immunotherapy [221]	++, 68.6%	Surgery, radiotherapy	30,2%, 52,4%, 17,4%	85.8%, 67.3%, 40.8%
Thyroid					
Papillary	Surgery, radioiodine therapy (^131^I), targeted therapy [222]	+++, 99.7%	Surgery, radioiodine therapy (^131^I)	63,6%, 33,8%, 2,7%	100.0%, 99.4%, 82.1%
Follicular	Surgery, radioiodine therapy (^131^I), targeted therapy [222]	+++, 94.3%	Surgery, radioiodine therapy (^131^I)	55,5%, 39,1%, 5,5%	99.1%, 93.2%, 33.8%
Medullary	Surgery, targeted therapy [222]	+++, 80.9%	Surgery	37,0%, 38,9%, 24,1%	100.0%, 92.2%, 24.4%
Anaplastic *	Surgery, radiotherapy, targeted therapy, chemotherapy [222]	+, 4.7%	Surgery	9,5%, 42,9%, 47,6%	0.0%, 11.3%, 0.0%
		Breast			
Breast	Surgery, chemotherapy, radiotherapy, targeted therapy, endocrine therapy [211]	+++, 90.8%	Surgery	63,2%, 29,3%, 7,5%	99.1%, 88.3%, 36.3%
Lung					
Non-small cell lung cancer	Surgery, radiotherapy, chemotherapy, immunotherapy, targeted therapy [223]	+, 21.0%	Surgery, radiotherapy [224] ^§^	18,3%, 24,1%, 57,6%	58.3%, 32.2%, 4.7%
Small cell lung cancer	Surgery, radiotherapy, chemotherapy, immunotherapy [225]	+, 7.0%	Surgery	19,3%, 80,4%, 0,3%	23.9%, 18.4%, 3.1%
Gastrointestinal cancer					
Oesophagus	Surgery, radiotherapy, chemotherapy, immunotherapy [226,227]	+, 22.3%	Surgery, endoscopic resection	21,4%, 32,6%, 45,9%	56.0%, 28.0%, 4.2%
Gastric	Surgery, radiotherapy, chemotherapy, targeted therapy, immunotherapy [42]	+, 20.2%	Surgery, endoscopic resection	22,5%, 28,9%, 48,5%	52.8%, 28.3%, 3.1%
Hepatocellular carcinoma	Surgery, intervention, targeted therapy, radiotherapy, chemotherapy, immunotherapy [228]	+, 21.2%	Surgical resection, transplantation, local ablation	53,4%, 31,6%, 15,0%	35.1%, 9.6%, 2.1%
Pancreatic cancer	Surgery, targeted therapy, intervention, radiotherapy, chemotherapy [229]	+, 8.4%	Surgery	21,0%, 78,9%, 0,1%	32.5%, 10.8%, 3.3%
Colorectal	Surgery, chemotherapy, targeted therapy, radiotherapy, immunotherapy [230]	++, 66.7%	Surgery, endoscopic resection	42,6%, 35,6%, 21,7%	90.2%, 75.0%, 13.6%
Anal	Surgery, chemotherapy, radiotherapy (EBRT, brachytherapy), targeted therapy [231]	++, 74.3%	Surgery	54,6%, 35,3%, 10,2%	83.7%, 67.9%, 44.7%
Genitourinary cancer					
Renal	Surgery, chemotherapy, targeted therapy, immunotherapy, ablation, radiotherapy [232]	++, 74.6%	Surgery, local ablation [174] ^§^	0,2%, 51,8%, 48,0%	92.9%, 70.0%, 11.4%
Bladder	Surgery, chemotherapy, targeted therapy, radiotherapy, immunotherapy [233]	+++, 77.3%	Surgery	0,3%, 77,9%, 21,8%	90.8%, 44.9%, 3.7%
Prostate	Surgery, chemotherapy, radiotherapy (EBRT, brachytherapy), endocrine therapy [210]	+++, 99.5%	Surgery	95,4% ^+,^ 4,6%	100% ^+,^ 28.2%
Testicular	Surgery, chemotherapy, radiotherapy [234]	+++, 96.4%	Surgery, chemotherapy, radiotherapy	71,5%, 17,9%, 10,6%	99.9%, 99.1%, 73.8%
Gynaecological cancer					
Ovarian	Surgery, chemotherapy, radiotherapy, targeted therapy, endocrine therapy [235]	++, 46.8%	Surgery	64,8%, 35,0%. 0,2%	95.0%, 62.8%, 31.7%
Endometrial	Surgery, chemotherapy, radiotherapy, targeted therapy, endocrine therapy, immunotherapy [236]	+++, 85.2%	Surgery	0,3%, 63,0%, 36,7%	96.5%, 71.6%, 32.5%
Cervical	Surgery, chemotherapy, radiotherapy (EBRT, brachytherapy), targeted therapy, immunotherapy [237]	++, 69.1%	Surgery	48,5%, 36,6%, 14,9%	92.4%, 60.9%, 18.0%
		Melanoma			
Melanoma	Surgery, chemotherapy, radiotherapy, targeted therapy, PDT, immunotherapy [238]	+++, 93.7%	Surgery, PDT, immunotherapy	0,7%, 68,6%, 30,7%	99.4%, 68.3%, 17.9%
Leukemia ^$^					
Acute lymphoid leukemia	Chemotherapy, targeted therapy, CAR-T, HSCT [239,240]	++, 74.0%	Chemotherapy, HSCT	NA	NA
Acute myeloid leukemia	Chemotherapy, targeted therapy, CAR-T, HSCT [241,242]	+, 28.6%	Chemotherapy, HSCT	NA	NA
Chronic lymphoid leukemia	Chemotherapy, targeted therapy, HSCT, observation ^¶^ [243]	+++, 82.2%	Chemotherapy, HSCT	NA	NA
Chronic myeloid leukemia	Chemotherapy, targeted therapy, HSCT, observation ^¶^ [244]	++, 70.0%	Chemotherapy, HSCT	NA	NA
Lymphoma ^£^					
Hodgkin’s	Chemotherapy, targeted therapy, HSCT, radiotherapy, immunotherapy [37]	+++, 86.7%	Chemotherapy, HSCT	15,4%, 42,6%, 22,1%, 20,0%	93.2%, 93.2%, 83.7%, 72.2%
Non-Hodgkin’s	Chemotherapy, targeted therapy, HSCT, radiotherapy, immunotherapy [36]	++, 72.6%	Chemotherapy, HSCT	28,1%, 16,6%, 17,5%, 37,8%	83.6%, 77.0%, 68.3%, 66.5%

Notes: Radiotherapy here refers to EBRT only, unless specified. Curative possibility (five-year RSR) scale: +—<30%; ++—30%-75%; +++—>75%. Abbreviations: RSR—relative survival rate; PDT—photodynamic therapy; HSCT—hematopoietic stem cell transplantation. ^‡^ All data here are accessed from the Surveillance, Epidemiology, and End Results (SEER) Program (www.seer.cancer.gov) SEER * Stat Database: Incidence—SEER 9 Regs Research Data, Nov 2018 Sub (1975–2016); The survival data by stage in anaplastic thyroid cancer is biased due to the few cases in each category. ^§^ Equivalent anticancer potential with surgery was only reported in retrospective studies but not in any randomized clinical trial. ^+^ Localized and regional prostate cancer cases together are merged as localized/regional cases. ^$^ All leukemia cases are categorized as distant cases and therefore stage distribution and corresponding survival information are blank. ^¶^ Patients with indolent cancer, based on a risk-stratification system, benefit more from active surveillance than any further intervention. ^£^ Lymphoma is staged based on Ann Arbor staging system.

## Abbreviations

ADCC	antibody-dependent cellular cytotoxicity
ALL	acute lymphoid leukemia
ALK	anaplastic lymphoma kinase
APC	antigen-presenting cell
CD	cluster of differentiation
CRC	colorectal cancer
CT	computed tomography
PET	positron emission tomography
CTLA-4	cytotoxic T-lymphocyte-associated protein 4
DFS	disease-free survival
DNA	deoxyribonucleic acid
EBRT	external beam radiotherapy
EGFR	epidermal growth factor receptor
EMA	European Medicines Agency
GI	gastrointestinal
HCC	hepatocellular carcinoma
HSCT	hematopoietic stem cell transplantation
ICIs	immune checkpoint inhibitors
IMRT	intensity-modulated radiation therapy
KRAS	Ki-ras2 Kirsten rat sarcoma viral oncogene homolog
MHC	major histocompatibility complex
MRD	minimal residual disease
NA	non-applicable
NCCN	national comprehensive cancer network
NP	nanoparticles
NPC	nasopharyngeal carcinoma
NSCLC	non-small cell lung cancer
OS	overall survival
PD-1	programmed death 1
PD-L1	programmed death ligand 1
PDT	photodynamic therapy
PFS	progression-free survival
PSA	prostate-specific antigen
RAI	radioactive iodine
RECIST	response evaluation criteria in solid tumours
R1	rhabdomyosarcoma
RSR	relative survival rate
SBRT	stereotactic body radiation therapy
SCLC	small cell lung cancer
SEER	surveillance, epidemiology and end results
TACE	transcatheter arterial chemoembolization
TCR	T cell receptor
TKI	tyrosine kinase inhibitor
TNM	tumour-node-metastasis
VDAs	vascular-disrupting agents
